# Influence of light/dark cycle and orexins on breathing control in green iguanas (*Iguana iguana*)

**DOI:** 10.1038/s41598-020-79107-2

**Published:** 2020-12-16

**Authors:** Elisa M. Fonseca, Mariane C. Vicente, Stephanie Fournier, Richard Kinkead, Kênia C. Bícego, Luciane H. Gargaglioni

**Affiliations:** 1 Department of Animal Morphology and Physiology, College of Agricultural and Veterinary Sciences, São Paulo State University, Unesp, Via de Acesso Prof. Paulo Donato Castellane s/n, Jaboticabal, SP CEP 14884-900 Brazil; 2 Department of Pediatrics, Institut Universitaire de Cardiologie et de Pneumologie de Québec, Université Laval, Quebec, QC Canada

**Keywords:** Physiology, Respiration, Animal physiology

## Abstract

Light/dark cycle affects the physiology of vertebrates and hypothalamic orexin neurons (ORX) are involved in this function. The breathing pattern of the green iguana changes from continuous to episodic across the light/dark phases. Since the stimulatory actions of ORX on breathing are most important during arousal, we hypothesized that ORX regulates changes of breathing pattern in iguanas. Thus, we: (1) Localized ORX neurons with immunohistochemistry; (2) Quantified cyclic changes in plasma orexin-A levels by ELISA; (3) Compared breathing pattern at rest and during hypoxia and hypercarbia; (4) Evaluated the participation of the ORX receptors in ventilation with intracerebroventricular microinjections of ORX antagonists during light and dark phases. We show that the ORX neurons of *I. iguana* are located in the periventricular hypothalamic nucleus. Orexin-A peaks during the light/active phase and breathing parallels these cyclic changes: ventilation is higher during the light phase than during the dark phase. However, inactivation of ORX-receptors does not affect the breathing pattern. Iguanas increase ventilation during hypoxia only during the light phase. Conversely, CO_2_ promotes post-hypercarbic hyperpnea during both phases. We conclude that ORXs potentiate the post-hypercarbic (but not the hypoxic)-drive to breathe and are not involved in light/dark changes in the breathing pattern.

## Introduction

The light/dark cycle is an important environmental signal that strongly affects physiological functions, including breathing^1–3^. In reptiles, breathing pattern can be continuous or intermittent^4,5^ and may change according to the light/dark cycle even at constant temperature^6,7^. In addition, the breathing pattern of these animals is influenced by high CO_2_ and low O_2_ concentrations^8^. Changes in these gases are detected by central (sensitive to pH and CO_2_), peripheral (sensitive to CO_2_, pH and O_2_) and intrapulmonary (CO_2_) chemoreceptors, as well as by olfactory and vomeronasal receptors in some species, adjusting respiratory pattern^9^. Most reptiles display a transitory increase in ventilation upon removal of CO_2_ and this post-hypercapnic hyperpnea arise from an interaction between the effects of CO_2_ acting on receptors at multiple sites. In fact, the post-hypercapnic hyperpnea appears to have evolved early amongst air-breathing vertebrates being demonstrated in air-breathing fish, amphibians and reptiles^8^. Furthermore, acid–base balance in ectotherms is determined by the interaction between temperature and time of the day^10^. Although these phenomena have been previously described, their underlying central mechanisms remain to be elucidated.

Orexins (ORXs, also called hypocretins) are neuropeptides implicated in the regulation of sleep/wake cycle and influence neuronal excitability during the light/dark phase^11^. Orexins include two neuropeptide subtypes, orexin-A (ORX-A) and orexin-B (ORX-B) (hypocretin-1 and hypocretin-2, respectively) both cleaved from a common precursor, prepro-orexin which binds to two G protein-coupled receptors: orexin receptor-1 (OX_1_R) and orexin receptor-2 (OX_2_R)^12^. Orexin receptor-1 is highly selective to ORX-A, whereas OX_2_R behaves as a nonselective receptor, binding to both ORX peptides with the same affinity^12^. Orexin levels have been measured in the cerebrospinal fluid of rodents, humans and monkeys^13^. These studies have shown that ORXs vary during the daily cycle, with the highest levels occurring during the active phase and the lowest levels during the inactive phase. Therefore, the discharge of ORX neurons is synchronized according to arousal states, with the highest activity taking place during active arousal^14^.

Orexins have an important phase-dependent role on breathing regulation^15–23^. For instance, in mice, ORX neurons increase their firing rate in response to increases in CO_2_/H^+^^21^ and genetic deletion of prepro-orexin attenuates the response to hypercapnia during wakefulness, but not during sleep phases. This effect is partially recovered with the administration of ORX-A and -B^15^. Besides that, intracerebroventricular administrations of an OX_1_R antagonist (SB-334867), decreased respiratory chemoreflex in rats^15,24^. The only study performed in a non-mammalian vertebrate shows that in toads *Rhinella diptycha* (former known as *R. schneideri*; Lavilla and Brusquetti^25^), a nocturnal amphibian, ORX-A acting on OX_1_R stimulates the hypercapnic chemoreflex during the dark phase. While ORX-A also stimulates the hypoxic chemoreflex, this influence is significant only during the light phase^26^. Therefore, despite considerable research regarding this neuropeptide, the participation of ORX in breathing control in non-mammalian vertebrates remains poorly characterized.

The amino acid sequences of ORX-A and -B are highly conserved across vertebrates^27^. Orexins are located in the hypothalamus and their distribution has been described in all classes of vertebrates; however, their organization is diffuse and not homogeneously confined within a specific area. Additionally, ORX fibers are widespread, innervating largely similar areas. In rodents, ORX neurons are found in the lateral and posterior hypothalamus and have widespread projections throughout the whole brain^28^. In amphibians, Galas et al.^29^ and Singletary et al*.*^30^ observed a single population of ORX neurons in the suprachiasmatic nucleus in *Pelophylax ridibundus* and *Hyla cinerea*, respectively. We also observed the same location for the toad *Rhinella diptycha*^26^ but conversely, Shibahara et al*.*^31^ demonstrated that ORX-containing neurons are present in the ventral hypothalamus in *Xenopus*. Regarding reptiles, most ORX immunoreactive neurons of lizards *Gekko gecko* and turtles *Trachemys scripta elegans* were found in the periventricular hypothalamic nucleus and in the infundibular hypothalamus^32^. Only in the gecko, ORX cell bodies were present in the dorsolateral hypothalamic nucleus and the periventricular preoptic nucleus as well. This anatomical property explains the great multiplicity of functions that are influenced by the ORXs, including the sensation of hunger, the sleep-awake state, feeding behavior, energy homeostasis, nociception, metabolism, the reward system, hormonal secretion, the response to stress, as well as the cardiovascular and respiratory control^33^.

Although the anatomical organisation of ORX neurons differs between species, the stimulatory actions of ORXs on breathing during the active phase seems highly conserved. With that in mind, we hypothesized that ORXs regulate phasic changes in the modulation of breathing pattern in green iguanas, a species which is active during daylight^34^. Testing this hypothesis addresses a novel concept in respiratory control of ectotherms and provides the first data on the organization and physiological role of the ORX system in reptiles.

## Results

### Localization of ORX neurons and daily variations of ORX-A in* Iguana iguana*

**Figure 1:**
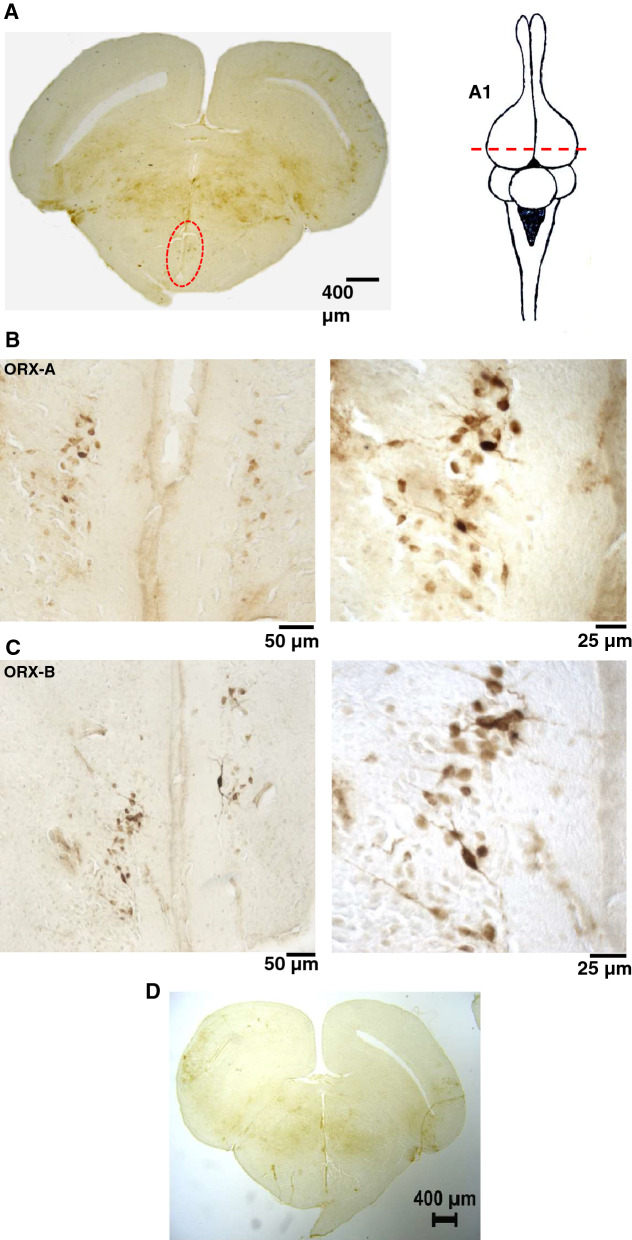
ORX labeling*. *Photomicrograph illustrating localization of the most prominent group of ORX-labeling and a scheme showing the level of the transection (**A**). The red circle indicates the localization of the cell bodies where were found the ORX neurons. A1: Schematic dorsal view of the lizard brain illustrating the rostro-caudal level where the section was taken. Photomicrographs illustrating ORX-A (**B**) and OXR-B labeling (**C**) in two different magnifications. Negative control (**D**).

The photomicrograph presented in Fig. 1A shows a frontal section at the rostro-caudal level where we found the largest number of ORX-ir cells in the hypothalamus of a representative iguana. The labeling observed was restricted to neuronal cell bodies and both ORX-A and ORX-B labeling was observed mainly in the periventricular hypothalamic nucleus, close to the ventricle. The labeling distribution in the brain was similar between ORXs-A and -B and no differences in the pattern of immunoreactivity were seen (Fig. 1B,C). Figure 1D shows a negative control.

**Figure 2:**
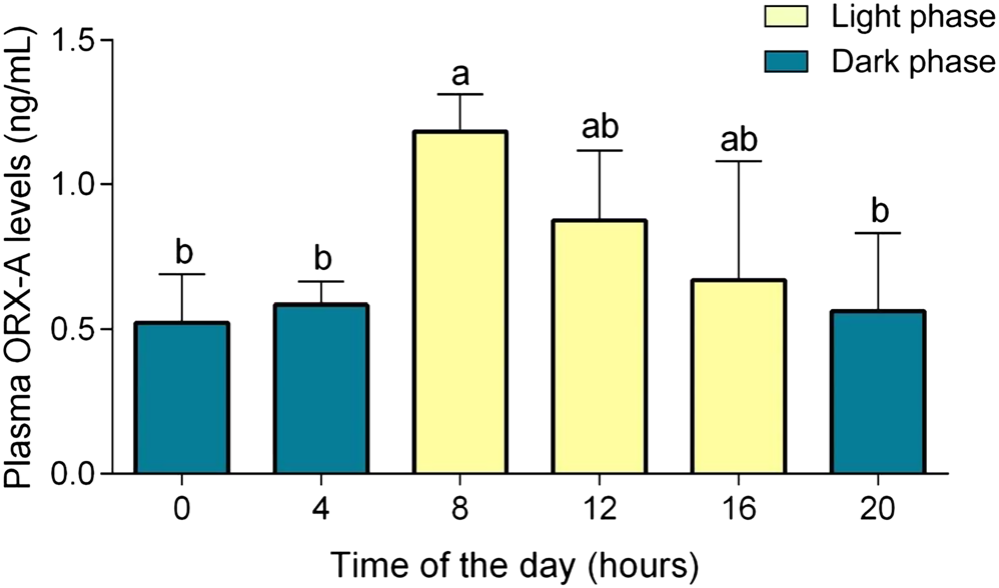
ORX-A plasma levels throughout the day. Variation during 24 h of the plasma levels of ORX-A in the green iguana (values represented as means ± s.e.m.). The yellow bars represent the light phase, while the blue bars represent the dark phase. Different letters mean significant statistical difference, and same letters mean that the difference is not statistically significant. Number of animals: 4 for each time of the day (total: 24).

Plasmatic ORX-A concentrations of the green iguana fluctuate across a day and peaked at 8:00 am (Fig. 2; 0 h vs 8 h: P < 0.0001, 4 h vs 8 h: P = 0.0007, 8 h vs 20 h: P < 0.0001, R^2^ = 0.8311, F (5, 18) = 17.72; different letters represent significant statistical difference).

### Differences in breathing along the day

**Figure 3:**
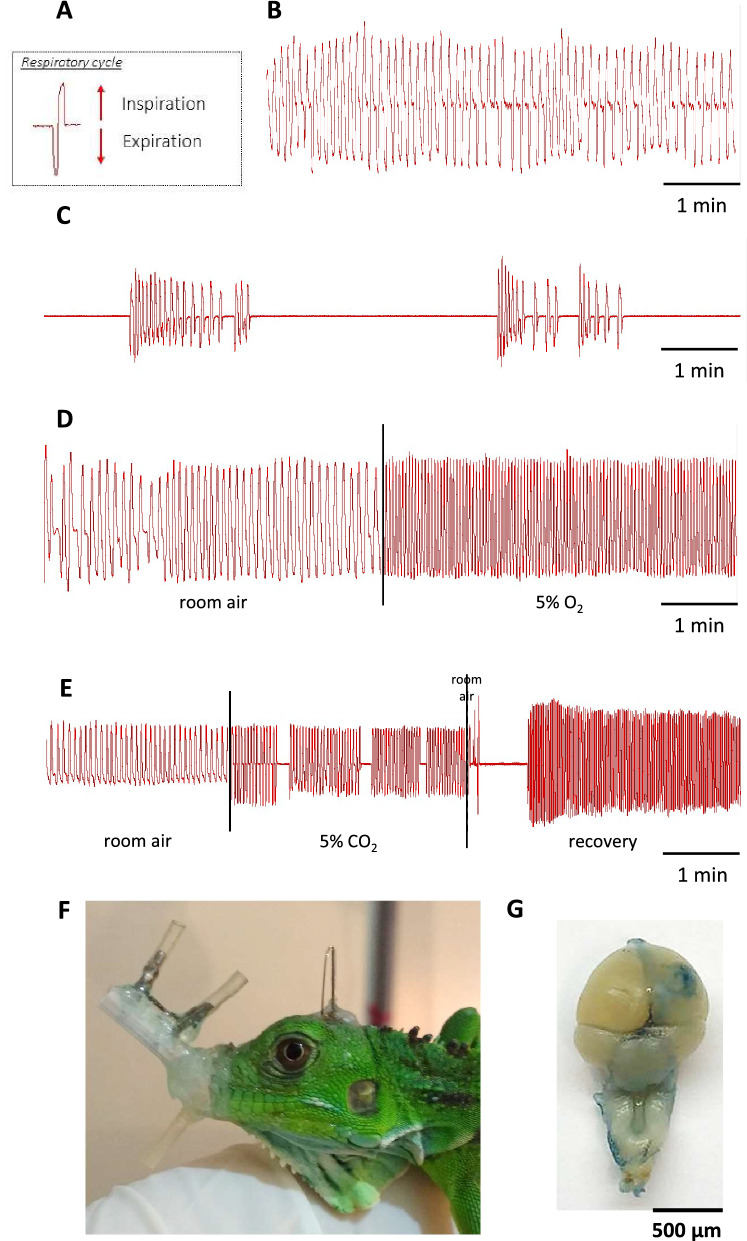
Representative pneumotachographic recordings of the different breathing patterns observed in *Iguana iguana*. Respiratory cycle (**A**). Representative traces from normocarbic normoxia (vehicle group) during light phase (**B**), normocarbic normoxia during dark phase (**C**), hypoxia 5% O_2_ during light phase (**D**) and hypercarbia 5% CO_2_ and recovery during light phase (**E**). The breathing pattern during the exposition to hypoxia or hypercarbia during the light and dark phases is similar, thus, only figures of the light phase are represented. Iguana with the pneumotachograph attached (**F**). Brain injected with Evans blue (**G**).

**Figure 4:**
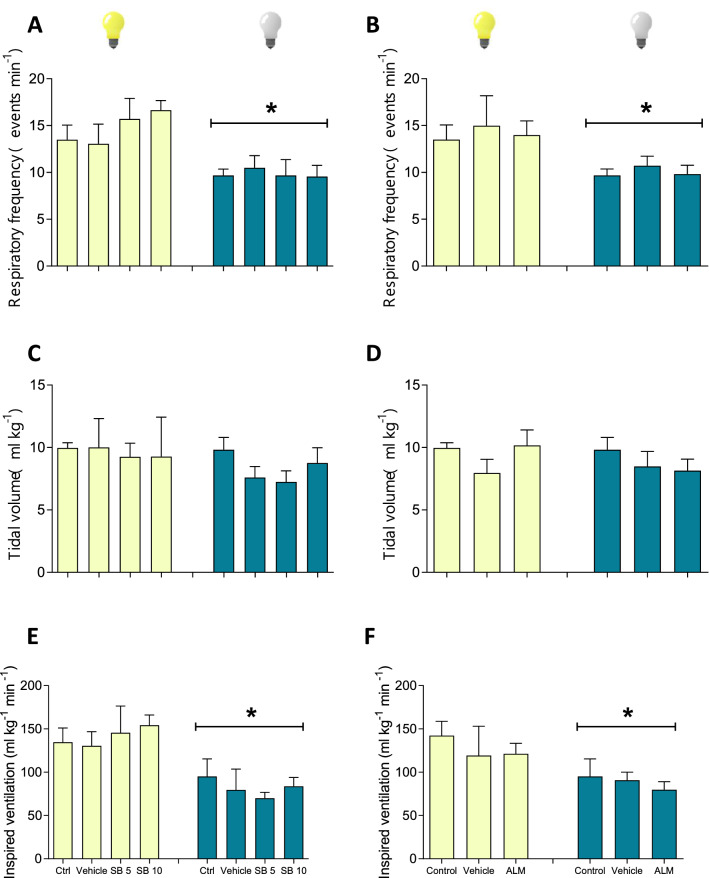
Effect of SB-334867 and Almorexant on basal ventilation. Effect of the i.c.v. injection of SB-334867 and its vehicle on fR, V_T_ and ${\dot{\text{V}}}_{{\text{I}}}$ in normocapnic normoxia in green iguanas during light or dark phases (**A**). Effect of the i.c.v. injection of Almorexant and its vehicle on fR, V_T_ and ${\dot{\text{V}}}_{{\text{I}}}$ in normocapnic normoxia in green iguanas during light or dark phases (**B**). * Means different from light phase at P < 0.05.

Before introducing data specific to the various experiments that addressed the participation of the ORX-receptors on ventilatory control, it is necessary to describe the diversity of the breathing pattern repertoire observed in *I. iguana* across the different experimental conditions considered in this study. Independent on the phase of the day, the respiratory cycle always started with an exhalation (Fig. 3A). During the light phase, resting iguanas breathed continuously (Fig. 3B) but in the dark phase, breathing became episodic and T_NVP_’s were intercalated within the ventilatory periods (Fig. 3C), making ${\dot{\text{V}}}_{{\text{I}}}$ smaller (Fig. 4E,F; P = 0.0165, F (1, 33) = 8.875). Thus, ${\dot{\text{V}}}_{{\text{I}}}$ was significantly greater during light phase due to a greater f_R_ (Fig. 4A,B; P = 0.004, F (1, 9) = 29.98). The phase of the day did not affect the V_T_ (Fig. 4C,D). Differences in the breathing pattern are shown in Table 1 and changes in ventilation can be seen in Table 2.

**Table 1:** Breathing pattern.

Treatment	Phase	Stimulus	TNVP ± s.e.m		Breaths/episode ± s.e.m		Episode freq ± s.e.m	
No injection	Light	Normoxia	–	–	–	–	–	–
	Dark	Normoxia	1.2	0.6	15.5	3.8	1.4	0.3
Vehicle SB-334867	Light	Normoxia	–	–	–	–	–	–
		Hypoxia	–	–	–	–	–	–
		Hypercarbia	0.8	0.3^+^	11.5	3.3	0.6	0.04
		Post-hypercarbia	–	–	–	–	–	–
	Dark	Normoxia	0.3	0.1	6.5	4.2	0.1	0.08
		Hypoxia	–	–	–	–	–	–
		Hypercarbia	2.5	0.8^+^	6.9	0.7	0.1	0.1
		Post-hypercarbia	–	–	–	–	–	–
SB-334867 5 μM	Light	Normoxia	–	–	–	–	–	–
		Hypoxia	–	–	–	–	–	–
		Hypercarbia	1.7	0.5	8.9	3.1	0.3	0.2
		Post-hypercarbia	–	–	–	–	–	–
	Dark	Normoxia	0.4	0.2	4.8	2.7	1.0	0.2
		Hypoxia	–	–	–	–	–	–
		Hypercarbia	2.8	0.4	3.4	1.4	0.1	0.1
		Post-hypercarbia	–	–	–	–	–	–
SB-334867 10 μM	Light	Normoxia	–	–	–	–	–	–
		Hypoxia	–	–	–	–	–	–
		Hypercarbia	1.3	1.1	6.8	3.4	0.4	0.2
		Post-hypercarbia	–	–	–	–	–	–
	Dark	Normoxia	0.5	0.3	3.9	2.9	1.8	0.9
		Hypoxia	–	–	–	–	–	–
		Hypercarbia	2.1	0.5	3.6	2.5	0.6	0.04
		Post-hypercarbia	–	–	–	–	–	–
Vehicle almorexant	Light	Normoxia	–	–	–	–	–	–
		Hypoxia	–	–	–	–	–	–
		Hypercarbia	0.8	0.3	9.3	3.2	0.4	0.1
		Post-hypercarbia	–	–	–	–	–	–
	Dark	Normoxia	0.3	0.2	6.4	3.2	0.1	0.05
		Hypoxia	–	–	–	–	–	–
		Hypercarbia	2.1	0.7	10.4	5.5	0.3	0.09
		Post-hypercarbia	–	–	–	–	–	–
Almorexant	Light	Normoxia	–	–	–	–	–	–
		Hypoxia	–	–	–	–	–	–
		Hypercarbia	1.8	0.3	2.2	0.7^#^	0.3	0.1
		Post-hypercarbia	–	–	–	–	–	–
	Dark	Normoxia	1.2	0.6	4.2	1.4	0.1	0.06
		Hypoxia	–	–	–	–	–	–
		Hypercarbia	4.1	1.1	2.7	0.6	0.2	0.08
		Post-hypercarbia	–	–	–	–	–	–

Means ± s.e.m. of T_NVP_ (min), number of breaths per episode and frequency of episodes (episodes min^−1^) of *I. iguana* microinjected with vehicle, SB-334687 or Almorexant exposed to room air, acute hypoxia or hypercarbia during light or dark phases. ^#^Means different from the vehicle and ^+^means different from normoxia.

**Table 2:** Ventilatory parameters.

Treatment	Phase	Stimulus	fR ± s.e.m		VT ± s.e.m		${\dot{\text{V}}}_{{\text{I}}}$ ± s.e.m	
No injection	Light	Normoxia	13.5	1.6	10.0	0.4	134.5	16.5
	Dark	Normoxia	9.7	0.7*	9.8	1.0	95.0	20.3*
Vehicle SB-334867	Light	Normoxia	13.0	2.1	10.0	2.3	130.4	16.3
		Hypoxia	20.5	1.1^+^	10.1	0.6	205.9	16.2^+^
		Hypercarbia	8.3	1.3	58.1	5.3^+^	484.7	83.7
		Post-hypercarbia	20.1	3.1^+^	51.6	4.5^+^	1035.7	200.3^+^
	Dark	Normoxia	10.5	1.3	7.6	0.9	79.6	24.0
		Hypoxia	14.7	1.8	7.9	1.6	115.9	28.1
		Hypercarbia	10.6	2.3	20.3	6.8	214.3	83.5
		Post-hypercarbia	15.4	2.0	23.2	7.8	356.3	86.5^+^
SB-334867 5 μM	Light	Normoxia	15.7	2.2	9.3	1.1	145.6	30.8
		Hypoxia	19.7	3.9	10.8	2.6	212.8	39.5
		Hypercarbia	11.3	1.3	27.6	4.6	312.3	79.0
		Post-hypercarbia	15.9	1.8	22.4	4.5^#^	357.2	98.6^#^
	Dark	Normoxia	9.7	1.7	7.2	0.9	69.8	6.7
		Hypoxia	15.2	1.7	9.5	1.0	144.9	17.1
		Hypercarbia	7.8	0.5	10.3	2.8	79.6	26.1
		Post-hypercarbia	14.6	0.5	21.4	5.5	312.9	72.1
SB-334867 10 μM	Light	Normoxia	16.6	1.0	9.3	3.2	154.3	11.8
		Hypoxia	15.7	3.2	8.3	3.5	131.1	41.1
		Hypercarbia	12.5	2.0	31.8	8.4	396.8	147.5
		Post-hypercarbia	15.5	3.7	27.5	6.5^#^	424.9	160.2^#^
	Dark	Normoxia	9.6	1.2	8.8	1.2	83.7	10.2
		Hypoxia	10.9	1.0	9.5	3.3	102.8	27.8
		Hypercarbia	8.4	0.6	8.4	0.4	70.3	6.0
		Post-hypercarbia	8.9	1.09	15.7	2.8	139.2	28.6
Vehicle almorexant	Light	Normoxia	15.0	3.2	8.0	1.1	119.3	33.8
		Hypoxia	21.6	1.6^+^	10.6	2.3	229.6	32.2^+^
		Hypercarbia	8.7	2.5	36.5	3.0	318.1	97.2
		Post-hypercarbia	21.9	1.3	46.1	9.7	1010.4	236.6
	Dark	Normoxia	10.7	1.0	8.5	1.2	90.8	9.4
		Hypoxia	10.5	1.1	9.1	2.1	94.9	24.9
		Hypercarbia	2.2	2.1	24.1	4.2^+^	53.9	19.5
		Post-hypercarbia	9.5	2.9^+^	28.4	7.7^+^	263.8	72.5^+^
Almorexant	Light	Normoxia	14.0	1.5	8.7	1.7	121.3	12.1
		Hypoxia	12.2	2.8	9.5	1.8	116.7	28.2
		Hypercarbia	7.6	0.5	19.1	9.3	145.1	86.5
		Post-hypercarbia	9.6	2.4^#^	32.3	7.1	310.5	27.3^#^
	Dark	Normoxia	9.8	0.9	8.1	0.9	79.8	9.2
		Hypoxia	7.6	1.6	7.7	1.5	58.4	11.2
		Hypercarbia	1.8	0.4	24.0	4.4	43.0	14.9
		Post-hypercarbia	7.4	0.9^#^	15.8	3.0	117.5	46.7^#^

Means ± s.e.m. of f_R_, V_T_ and ${\dot{\text{V}}}_{{\text{I}}}$ of *I. iguana* microinjected with vehicle, SB-334687 or Almorexant exposed to room air, acute hypoxia or hypercarbia during light or dark phases. Statistical differences are represented on the graphs. * Means different from the light phase; ^#^means different from the vehicle and ^+^means different from normoxia.

### Reflex responses

**Figure 5:**
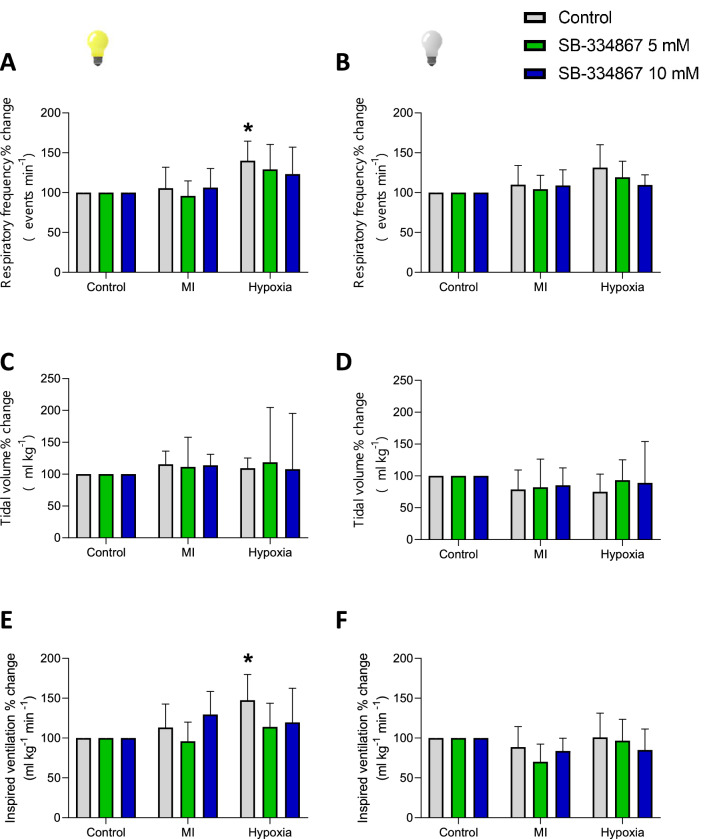
Effect of SB-334867 on hypoxic chemoreflex. Effect of the i.c.v. microinjection (MI) of SB-334867 or its vehicle on f_R_, V_T_ and ${\dot{\text{V}}}_{{\text{I}}}$ in green iguanas exposed to acute hypoxia (5% O_2_) during light or dark phases. Data reported as means ± s.e.m. The effect of hypoxia (vehicle group, no drug) is indicated by * compared to control, while + represent the statistical differences caused by the effect of the drug.

During hypoxia, breathing pattern was continuous (Fig. 3D), and a small but significant increase in ${\dot{\text{V}}}_{{\text{I}}}$ was observed during the light phase (Table 2; P = 0.0214; R^2^ = 0.6690; F (1.566, 6.264) = 8.085) due to an increase in the f_R_ (P = 0.0098; R^2^ = 0.7408, F (1.565, 6.262) = 11.43). Figure 3D shows a representative trace of the hypoxic ventilatory response compared to normoxic air. Conversely, the green iguanas did not respond to acute hypoxia during the dark phase (Fig. 5, Tables 1 and 2).

**Figure 6:**
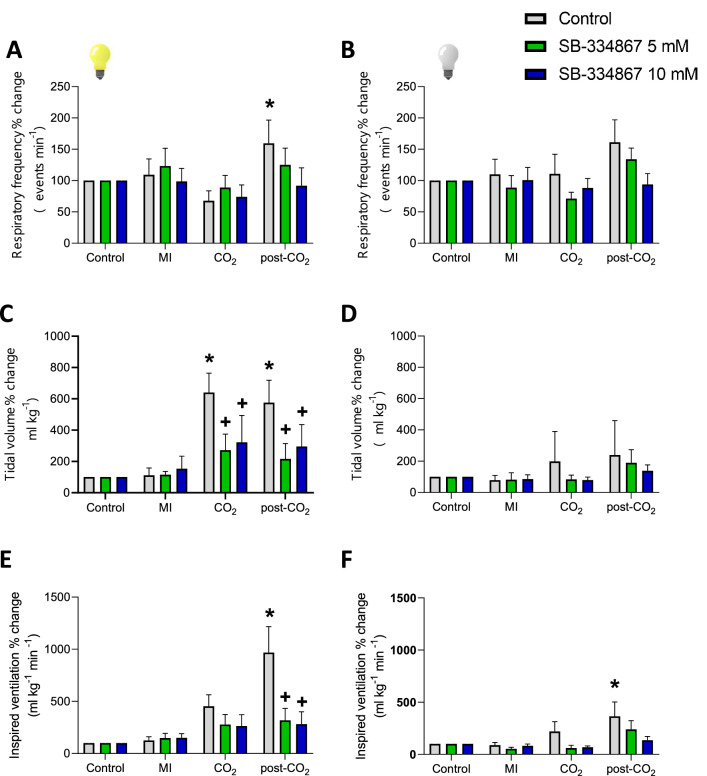
Effect of SB-334867 on CO_2_-chemoreflex. Effect of the i.c.v. microinjection (MI) of SB-334867 and its vehicle on f_R_, V_T_ and ${\dot{\text{V}}}_{{\text{I}}}$ in green iguanas exposed to acute hypercarbia (5% CO_2_) during light or dark phases. Data reported as means ± s.e.m. * means different from vehicle. The effect of hypercarbia (vehicle group, no drug) is indicated by * compared to control, while + represent the statistical differences caused by the effect of the drug.

Figure 3E illustrates what happened when iguanas were exposed to hypercarbia. In the light phase, during the exposure, the f_R_ lightly decreased while the V_T_ lightly increased resulting in a balanced effect that did not change the ${\dot{\text{V}}}_{{\text{I}}}$. During CO_2_, breathing pattern was episodic independent on the phase (Fig. 3E; Table 1). Once the CO_2_ was removed, ${\dot{\text{V}}}_{{\text{I}}}$ increased abruptly and the breathing pattern became continuous, thus revealing the post-hypercarbic hyperpnea (P = 0.0004, R^2^ = 0.6669, F (3, 16) = 3.811) due to an increase in f_R_ (P = 0.0020; R^2^ = 0.5930; F (3, 16) = 7.772) and V_T_ (P = 0.0005; R^2^ = 0.6559; F (3, 16) = 10.17). During the dark phase, V_I_ did not increase during hypercarbia, but a similar post-hypercarbic hyperpnea response was observed; however, this response was smaller to the one observed during the light phase (Fig. 6F; P = 0.0737, R^2^ = 0.5142, F (3, 16) = 2.797).

Regarding the breathing pattern (Table 1), mean data support observations made on the original recordings and show that during light phase, 5% CO_2_ changed the breathing pattern from continuous to episodic by increasing the T_NVP_ (P = 0.0456; R^2^ = 0.4058; F (3, 16) = 2.510). During the dark phase, the resting pattern was already episodic. During post-hypercarbia and hypoxia, breathing was always continuous, and the pattern remained constant (Table 1).

### Effect of ORX antagonists on the breathing pattern

Table 1 reports that the specific OX_1_R antagonist SB-334867 had no influence on breathing pattern variables compared to vehicle either in room air, hypoxia or hypercarbia (and post-hypercarbia) during light or dark phases. Conversely, the non-selective (OX_1_R and OX_2_R) antagonist decreased the number of the breaths per episode during 5% CO_2_ exposure during the light phase (P = 0.0220; F (1, 36) = 5.733). Almorexant had no other influence on breathing pattern.

### Effect of SB-334867 on ${\dot{\text{V}}}_{{\text{I}}}$

During basal conditions, microinjection of SB-334867 either at 5 or 10 µM had no effect on the breathing pattern or ${\dot{\text{V}}}_{{\text{I}}}$ independent on the phase (Fig. 4; Tables 1 and 2). Regardless the dose, the antagonist did not alter the O_2_ chemoreflex in green iguanas during either phase (Fig. 5).

By contrast, during the light phase, SB-335867 did not affect the ventilatory response to CO_2_ but both doses of SB-334867 attenuated the post-CO_2_ hyperpnea during both phases (Fig. 6; light phase: F (2, 48) = 4.492; P < 0.0001 for SB 5 µM and P = 0.0001 for SB 10 µM; dark phase: F (2, 48) = 3.217; P = 0.0036 for SB 5 µM and P = SB 10 µM). This decrease was mainly due to a reduced V_T_ (F (2, 52) = 20.67; P < 0.0001 for 5 and 10 µM). During the dark phase, however, neither dose of SB-334867 affected the hypercarbic chemoreflex nor the post-hypercarbic hyperpnea (Fig. 6; Table 2).

### Effect of Almorexant on ${\dot{\text{V}}}_{{\text{I}}}$

**Figure 7:**
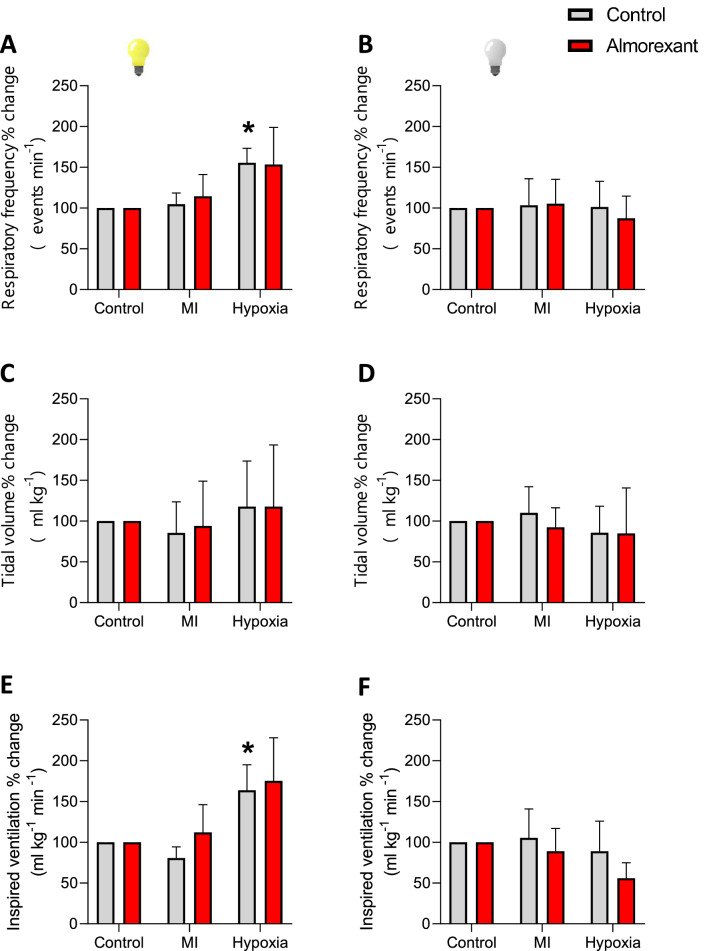
Effect of Almorexant on hypoxic chemoreflex. Effect of the i.c.v. microinjection (MI) of Almorexant and its vehicle on f_R_, V_T_ and ${\dot{\text{V}}}_{{\text{I}}}$ in green iguanas exposed to acute hypoxia (5% O_2_) during light or dark phases. Data reported as means ± s.e.m. The effect of hypoxia (vehicle group, no drug) is indicated by * compared to control, while + represent the statistical differences caused by the effect of the drug.

Treatment with this dual ORX antagonist did not affect breathing under basal conditions or hypoxia during either phase (Figs. 4, 7; Table 2).

**Figure 8:**
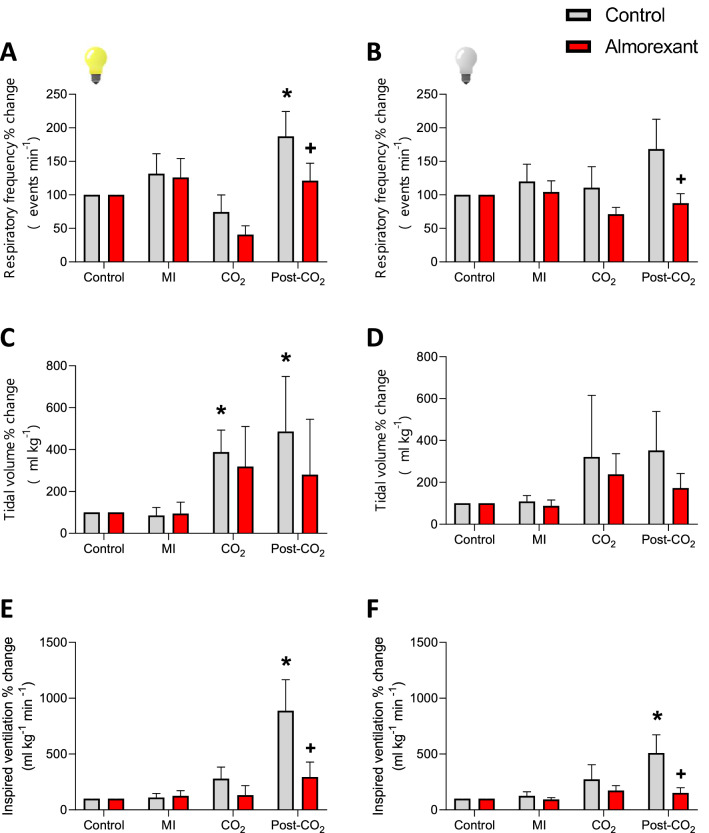
Effect of Almorexant on CO_2_-chemoreflex. Effect of the i.c.v. microinjection (MI) of Almorexant and its vehicle on f_R_, V_T_ and ${\dot{\text{V}}}_{{\text{I}}}$ in green iguanas exposed to acute hypercarbia (5% CO_2_) during light or dark phases. Data reported as means ± s.e.m. * means different from vehicle. The effect of hypercarbia (vehicle group, no drug) is indicated by * compared to control, while + represent the statistical differences caused by the effect of the drug.

Ventilatory response to CO_2_ in this group was in line with results obtained in the previous group. Almorexant attenuated ${\dot{\text{V}}}_{{\text{I}}}$ during the post-hypercarbic phase during the light and dark phases (P = 0.0001; F (1, 36) = 9.054 for light phase and P = 0.0085; F (1, 32) = 8.139 for dark phase; Fig. 8; Table 2). This decrease was due to reduction in the f_R_ response (P = 0.0014, F (1, 36) = 15.89) for light phase and for the dark phase, although the decrease in the fR was not statistically significant, the combining effect of the decrease in the f_R_ with the decrease in V_T,_ promoted the attenuation of ${\dot{\text{V}}}_{{\text{I}}}$.

## Discussion

The green iguana is a diurnal animal^34^ and the day-night differences in respiratory activity and ORX modulation reported here reflect this condition. We show that inactivation of ORX receptors attenuates reflexive responses to respiratory stimuli (especially to CO_2_ during light phase). Contrary to our initial prediction, none of the pharmacological treatments tested here influenced breathing pattern at rest. While these results do not support our main hypothesis, they nonetheless provide new valuable insight into respiratory control in this species. This is the first study showing the contribution of ORXs to the respiratory control in a reptile and in a diurnal animal.

The ORX neurons of *Iguana iguana* are located in the hypothalamus, more specifically in the periventricular hypothalamic nucleus. According to Volkoff^13^, the structures of the ORX genes, peptides, and receptors are conserved among vertebrates, suggesting that their physiological functions are also similar. The neuroanatomical distribution of ORX neurons appears to be also well conserved, with little species-specific differences. In our study, we found distinct somatic staining of ORX-ir cells within the hypothalamus, more specifically in the periventricular hypothalamic nucleus. We could not see much innervation in the brain, but the staining on the cell bodies are strong and consistent. It is also in accordance to other studies performed in reptiles^32,35^.

Only a few studies have examined the distribution of ORXs within the reptilian brain. Studies performed in the lizard *Anolis carolinensis*, in the lizard *Gekko gecko* and in the turtle *Trachemys scripta elegans* showed ORX-ir neurons in the periventricular and the infundibular hypothalamus and ORX-ir fibers are extensively distributed in the whole brain^32,35^. In the gecko and in the turtle, ORX innervation of respiratory areas such as the *locus coeruleus*, the nucleus of the solitary tract and the raphe nuclei regions has been reported^32^. While the physiology of ORXs in reptiles has received limited attention, the distribution of ORX fibers within the reptilian brain suggests a role for these peptides in functions like energy homeostasis, arousal and breathing, as is well known in mammals^13^.

The metabolic rate of ectotherms changes as a function of temperature^36^. Thus, to attenuate changes in ventilation as a consequence of the metabolism, ventilation was measured at a set temperature in both phases. The temperature of 25 °C represents the preferred body temperature during the dark phase and to which the animals were already acclimated. While this temperature differs from the daily peak, it was still possible to observe the differences in breathing and chemoreflexes between the light and dark phases. This approach allowed us to assess the participation of ORXs in the modulation of respiratory control while eliminating the impact of temperature as a confounding factor.

In the present study, we discerned differences on the respiratory parameters of the green iguanas between the light and dark phases. Specifically, we showed that ${\dot{\text{V}}}_{{\text{I}}}$ is greatest during the light phase, due to a greater f_R_; this observation parallels plasma ORX-A which are greater during that phase and peak near 8 a.m. In the tegu (*Salvator merianae*), in red-eared sliders (*Trachemys scripta*), box turtles (*Testudo pardalis*) and garter snakes (*Thamnophis elegans*), all diurnal reptiles, nighttime reductions in ventilation under natural conditions were also achieved primarily by decreasing fR, not V_T_^1,7,37^. These studies show that ventilation presents a daily oscillation that seems to follow the level of activity.

The reduction in fR was operated by changing the pattern from continuous to breathing in episodes during the night, leading to long T_NVP_. This is consistent with the observation that the duration of the T_NVP_ is the primary variable regulated in the breathing pattern of reptiles^38^. These daily changes in ventilation and breathing pattern likely reduce the costs of ventilation and blood gases transport^39^. Further, according to the same study, day-night differences in ventilation resulted from changes in chemoreflex sensitivity.

In the current study, the microinjection of ORX antagonists (SB-334867 and Almorexant) during both phases of the daily cycle did not promote any changes in ${\dot{\text{V}}}_{{\text{I}}}$ or breathing pattern under room air conditions in *I. iguana,* suggesting that central ORX does not play a tonic respiratory role. The literature is scarce in studies showing the role of ORXs in ventilatory control in non-mammalian vertebrates; the only one is a study from our group performed in toads that shows also that SB-334867 did not promote any changes in ${\dot{\text{V}}}_{{\text{I}}}$ under normocarbic normoxia^26^. In mammals, ORX neurons project directly to respiratory neurons in the brainstem and these structures express ORX-receptors^40^. Similar to our results, the focal antagonism of OX_1_R in the RTN^24^ or the rostral medullary raphe^41^ does not influence spontaneous ventilation in rats. Moreover, the i.c.v. administration of SB-334867 in mice does not change ventilation in room air during either wakefulness or sleep^15^. On the other hand, there are studies showing that ORXs stimulate breathing in rodents^42,43^.

Based on these observations, it is plausible that ORX neurons of iguanas behave similarly to mammals^14,44^ and show little activity at rest (during room air) and consequently activation of ORX-receptors is limited under those conditions. Nevertheless, as our experimental protocol did not assess the activity of the ORX neurons, further experiments are necessary to prove this hypothesis better. Results from pharmacological experiments show that inactivation of ORX receptors attenuated the response to CO_2_. The fact that the dual antagonist attenuated mainly the frequency response whereas the selective ORX-1 antagonist was more effective at reducing the V_t_ response points to different effects on mechanisms regulating V_T_ versus f_R_. This may reflect differences in the distribution of ORX-receptors and/or differences in the availability of ORXs among multiple respiratory centers or the role of each area in controlling V_T_ or f_R_. Therefore, our findings, taken with other evidence in the literature, suggest that in this reptile, central ORX does not play a tonic respiratory role, but it may be important in specific situations, such as hypercarbia and hypoxia.

Most animals increase pulmonary ventilation when breathing hypoxic gas mixtures^45^. Acute hypoxia usually increases ventilation^46^, but a reduction in ventilation has been also reported^47^. Here, hypoxia increased ${\dot{\text{V}}}_{{\text{I}}}$ during the light phase, but not during the dark phase; this observation is in line with the daily differences in central and peripheral-chemosensing discussed previously^1,2,48–51^. In addition, ventilation during hypoxia was continuous during both phases, what agrees with the usual response to hypoxia that is a shortening of the T_NVP_ with or without an increase fR or V_T_^5^. Additionally, the green iguana seems to be less sensitive to changes in ambient PO_2_ than to variations in PCO_2_ such as other species of lizards like *Tropidurus torquatus*, *Lacerta sicula*, *Lacerta viridis* and *Uromastyx aegyptius microlepis*^8,52,53^.

Regardless of the light/dark cycle, injection of SB-334867 or Almorexant did not change the ventilatory response to hypoxia or the breathing pattern of iguanas. In contradiction, in the other single study aforesaid performed on a non-mammalian vertebrate, toads from the genus *Rhinella* present an attenuated ventilatory response to hypoxia during the light, but not during the dark phase when microinjected with SB-334867^26^. Our results agree with those obtained in mammals also using a similar approach^15^. Previous study has demonstrated that the hypoxic response of prepro-orexin knockout mice does not differ from that of wild-type mice^18^, thus, suggesting that ORX is not involved in the peripheral chemoreflex in mammals. Although the literature is quite diverse, our results show that in the iguana, ORXs do not contribute to the O_2_-chemoreflex.

As to CO_2_ challenge*,* the response to hypercarbic concentrations in reptiles is variable. Apparently, high inspired CO_2_ concentrations are tolerated well by reptiles and result in only minor increases in ventilation^5,37,46^. Central chemoreceptors are an important source of respiratory drive and their functionality has been established in reptiles^54–58^. Exposing green iguanas to 5% CO_2_ evoked no changes in ${\dot{\text{V}}}_{{\text{I}}}$, but in the respiratory pattern during the stimulation period, and a robust post-CO_2_ hyperpnea upon return to room air, an off-response observed also in other lizard species^8,53,59^. When the inhibition of the CO_2_-sensitive airway chemosensors is removed, then peripheral and central chemoreceptor stimulate ventilation. Both components of the response were greater during the light phase rather than during the dark phase.

The greater post-hypercarbic response during the light phase agrees with data obtained in red-eared sliders showing a reduced respiratory response to the hypoxic/hypercarbic stimulus at night compared with the day^2^. Time of the day affects the ventilatory response to hypoxia, hypercapnia or both stimuli combined in mammals^1,49,50^ and birds^51^. Thus, it is possible that the cyclic changes observed in response to CO_2_ in this study are due to the cyclic changes seen in the ORX levels along the day. This oscillation might affect the modulation of the CO_2_-chemoreflex, but more experiments are necessary to affirm.

About the participation of ORX-receptors in the CO_2_-chemoreflex, the central microinjection of SB-334867 or Almorexant caused an attenuation of the ventilatory response to the post-CO_2_ exposure due to decreases in the fR for the Almorexant, and in the V_T_ for the SB-334867. We observed that OX_1_R antagonism attenuated the post-CO_2_ response of iguanas only during the light phase, while the antagonism of both receptors by Almorexant had a similar effect during both phases. These data suggest that ORXs acting on OX_1_R and OX_2_R in the CNS are important modulators of the central chemoreflex in green iguanas, but their relative role may differ between day and night. The post-hypercapnic hyperpnea is possibly mediated by removal from inhibitory influence of airway chemoreceptors reflecting the “true” effect of systemic hypercapnia on peripheral and central chemoreceptors^60^. Therefore, ORX system may affect the central or peripheral chemoreflex to increase the post-hypercapnic response to CO_2_. We suggest that central chemoreceptors are the most putative sites to be affected, since ORX antagonist did not affect hypoxic ventilatory response. The fact that no effect of ORX antagonism on ventilation was observed during CO_2_ exposure is possibly due to the activation of inhibitory input from the airway receptors.

Our data agree with previous reports in mammals in which by different approaches, results show that ORXs contribute to the CO_2_-chemoreflex in an excitatory way, more importantly during the active phase^15,41^. Moreover, in a non-mammalian vertebrate, SB-334867 promoted an attenuation on the ventilatory response to hypercarbia (5% CO_2_) in *Rhinella* toads (a nocturnal animal) during dark, but not during the light phase. Conclusively, ORXs potentiate the CO_2_-drive to breathe and it seems like this modulation is well conserved across vertebrates (at least the ones that we have the data so far). It is possible that for animals that are dependent of the aquatic environment like the amphibians, for which changes in O_2_ are limiting to trigger changes in respiratory parameters, ORXs participate on the O_2_-chemoreflex; but in animals completely adapted to the terrestrial environment, like lizards and mammals, that are more sensitive to the changes in CO_2_, ORXs are more important on the CO_2_-chemoreflex. Nonetheless, to have a better understanding of how ORXs modulate ventilation across the groups of vertebrates more studies are necessary, especially with other groups not yet studied.

In summary, our results demonstrate that: (1) ORX neurons are located in the periventricular hypothalamic nucleus in *Iguana iguana*; (2) these animals are diurnal and have higher ORX-A levels during early morning; (3) ORXs do not modulate breathing pattern; (4) ORXs—acting on OX_1_Rs and OX_2_R—contribute to the post-hypercarbic hyperpnea but not to the hypoxic chemoreflex in green iguanas. The present observations, taken together with other studies, indicate a considerable degree of phylogenetic conservation of the orexinergic pathway among vertebrates.

## Material and methods

### Animals

Juvenile green iguanas (*Iguana iguana*) of either sex weighing 44.8 ± 2.1 g were obtained from the Jacarezário of the São Paulo State University, campus of Rio Claro. The animals were transported and maintained in agreement with SISBIO-ICMBio (animal license 50041) in the Department of Animal Morphology and Physiology of the São Paulo State University in the campus of Jaboticabal, where the experiments were performed. This study was conducted in compliance with the guidelines of the National Council for Animal Experimentation Control (CONCEA). The experimental protocol was approved by our institutional Animal Care and Use Committee (CEUA-FCAV-UNESP 3060/16). The iguanas were maintained in a room at 25 °C in large tanks with shavings and twigs mimicking perches. Warming lamps and UV illumination remained on throughout the light phase (during 12 h).

Two groups of iguanas were kept in different light/dark cycles: one group was maintained under natural light/dark cycle; the other was kept under a displaced, artificial light/dark cycle [lights turned on at 00:00 (midnight) and turned off at 12:00 (noon)]. The iguanas kept under the natural cycle were used for the ELISA assays, immunohistochemistry and the physiology (ventilation) experiments performed during the light phase. The iguanas maintained under the displaced cycle were used for the second part of the ventilation experiments performed during the dark phase.

### Series I: Evaluation of the orexin system in the green iguana

#### Localization of ORX neurons in green iguana

Immunohistochemistry was performed to verify the localization of ORX neurons in this species. Orexin-A and ORX-B labeling were conducted separately. This protocol was used for qualitative purposes only and were performed according to a previous study^35^. The four intact animals used for those experiments were not used in other protocols. The iguanas were anesthetized by intraperitoneal pentobarbital injection (Thiopental; Cristalia, Brazil) and perfused through the heart with PBS (0.01 M, pH 7.4). Once the blood was cleared, perfusion was continued with phosphate buffer (0.01 M PB, pH 7.4) containing 4% paraformaldehyde (PFA). The brains were dissected, post-fixed with the same fixative solution for 4 h and stored in 30% sucrose in PBS at 4 °C overnight. Then, the tissues were placed in an embedding medium (Tissue Tek, Germany) and immediately frozen in isopentane and sliced into 30 µm sections using a cryostat (CM 1850; Leica, Germany). Before starting the labeling, the slices were incubated in a retrieval solution (Target Retrieval Solution Ready-to-use, Dako, Denmark) at 70 °C (in water bath in microtubes) for 30 min.

The sections were incubated for 48 h with a rabbit polyclonal anti-ORX-A (1:500; Santa Cruz, USA) or anti-ORX-B (1:500; Santa Cruz, USA) antibodies followed by a 2 h incubation with a biotinylated goat polyclonal anti-rabbit IgG (1:1000; Vector Laboratories, USA) antibody. The biotinylated antibody was complexed with avidin biotinylated horseradish peroxidase (PK-4001; Vector Laboratories, USA), and the complex was developed by addition of the peroxidase substrate 3,3-diaminobenzidine tetrahydrochloride according to manufacturer instructions (DAB; Sigma-Aldrich, USA). The reaction was terminated by washing out excessive amounts of PBS (phosphate buffer solution, pH 7.4 at 25 °C). Finally, the sections were mounted on gelatin-coated slides, dried, dehydrated through graded concentrations of alcohol, cleared in xylene, and sealed with a coverslip. Photomicrographs of the brain were captured by an optic microscope (Leica, Germany) using the image acquisition software LAS (Leica, Germany). We included a negative (without primary antibody) control to test the specificity of the immunohistochemistry protocol.

#### Circadian variation of ORX-A

In a distinct group of iguanas (n = 24), we determined whether ORX-A levels fluctuate throughout the daily cycle by measuring plasma levels of ORX-A with an ELISA assay. These animals were maintained in our animal facility with a standard daily cycle (natural photoperiod, no cycle inversion). After one year of acclimation in our research facilities, these iguanas were used for the light phase of the ventilation experiments and blood collection. ELISA kit Orexin A (Extraction Free EIA Kit; Phoenix Pharmaceuticals, USA) was used and the tests were performed according to the company’s protocol. Phoenix Pharmaceutical states that the minimum detection limit of ORX-A is 0.22 ng/mL. The ELISA has 100% cross-reactivity with iguanid ORX-A. There was no cross-reactivity of the antibody for other neuro-hormones. The intra-assay error < 10%, and the inter-assay error < 15%. The iguanas were anesthetized with isoflurane (Cristalia, Brazil) and terminal blood samples were collected from the heart from different animals at 6 different time points (4-h interval): 00:00, 04:00, 08:00, 12:00, 16:00, and 20:00. Iguanas sampled during the dark phase were anesthetized very quickly, and covered in a heavy black cloth during blood collection; the collections procedure for this phase were carried out with very little light. The blood was collected with a heparinized syringe, homogenized with aprotinin (Sigma-Aldrich, USA), centrifuged and the plasma was kept at − 80 °C until ELISA analysis.

For each time point, 4 iguanas were used and the samples were analyzed in duplicate (totalizing 24 iguanas in 6 time points and 48 wells). We ran two assays, so two plates were used for this protocol.

### Series II: Role of ORX in light/dark phase differences in respiratory control

These experiments aimed to (1) determine if the light/dark cycle affects breathing pattern and reflexive responses to ventilatory stimuli (↓O_2_ and ↑CO_2_) and (2) evaluate the differential role of ORX receptors in respiratory control across the light/dark cycle. To do so, all animals were instrumented with an intracranial cannula allowing intracerebroventricular injection of either vehicle or an ORX antagonist over the course of ventilatory measurements.

#### Stereotaxic surgery

Animals were anesthetized with isoflurane (1–1.5%). The heads of the animals were then fixed in a stereotaxic apparatus (Model 900 Small Animal Stereotaxic; David Kopf, USA), the skin covering the skull was removed using a bone scraper, and an opening was made in the skull above the telencephalon using a small drill (LB100; Beltec, Brazil). For microinjection, a guide cannula prepared from a hypodermic needle segment of 12 mm in length and 0.55 mm in outer diameter was attached to the tower of the stereotaxic apparatus and placed into the lateral cerebral ventricle. These coordinates were adapted according to the brainstem atlas for the lizard *Varanus exanthematicus*^61^. During cannula implantation, care was taken to avoid damaging or covering the modified transparent scale of the “parietal eye”. This “eye” is a photoreceptive structure associated with the pineal gland that contributes to the regulation of circadian rhythmicity. The displacement of the meniscus in a water manometer confirmed the correct positioning of the cannula within the lateral ventricle. The orifice around the cannula was filled out with a wax made of equal parts of paraffin and glycerin. The cannula was attached to the bone with acrylic cement mixed to superglue. A tight-fitting stylet was kept inside the cannula to prevent occlusion and infection. After stereotaxic surgery, the iguanas were treated with prophylactic antibiotic (5.0 mg/kg, intramuscular; Enrofloxacin, Flotril; Schering-Plough, USA) and nonsteroidal anti-inflammatory (0.2 mg/kg, subcutaneous, Meloxicam; Eurofarma, Brazil) agents according to recommended doses for reptiles for three days^62,63^. The surgery and postoperative care protocols were designed by the veterinarian responsible for the animal care of the university. The operated iguanas were monitored after surgery and at any sign of pain, the veterinarians were called in to check the animals. Animals recovered from brain surgery at least 7 days before experiments were initiated.

#### Instrumentation for ventilatory measurements

Pulmonary inspired ventilation (${\dot{\text{V}}}_{{\text{I}}}$), tidal volume (V_T_), and breathing frequency (fR) were measured using the pneumotachograph method^37^, which is based on the Poiseuille principle that a laminar flow of a gas is proportional to the pressure gradient across a tube. As represented by Fig. 3F, a lightweight small transparent facemask attached to a pneumotachograph (made with plastic tubes) was fixed to the animal snout, allowing for inspirations and expirations to be measured continuously. The mask was installed with two-part epoxy adhesive at least 6 h before the beginning of the experimental protocol; this procedure was not invasive and did not require anesthesia. The animal could move freely with the pneumotachograph on and the mask could be easily removed after the experiment. Inspiratory and expiratory gas flows were monitored with a differential pressure transducer connected to a data acquisition system that included specific application software (MLT141 Spirometer, Power Lab: ADInstruments/LabChart Software, Australia). Calibration for volume was performed after each experiment by injecting known volumes (0.5–5 mL) of air using a graduated syringe into the facemask glued to a plaster mold (we used a mold for each animal). All measurements of pulmonary ventilation were performed at a constant temperature: 25 °C. This temperature was chosen based on the temperature of the cages during the dark phase (lights off). The preferred body temperature of the green iguana fluctuates around 34 °C (active/light phase) and 25 °C (inactive/dark phase) depending on the temperature of acclimatization^64–66^. As the purpose of the study is to investigate the effects of the phase on breathing and if ORXs contribute to this modulation; we chose one single temperature to perform the ventilation experiments, considering that in ectotherms, metabolism is directly influenced by temperature^36^. This approach allows us to determine whether the observed effects are due to the phase and the ORX-neurotransmission and not due to thermoregulatory cues.

#### Intracerebroventricular microinjections of ORX antagonists

Experiments were performed on two major groups of iguanas: 24 animals for the measurements of the light phase (also used for blood collection) and 24 animals for the dark phase (displaced light/dark cycle). Within these two major groups, experiments were performed on three subgroups of iguanas: group I received the vehicle (n = 5); group II received the selective ORX-1 receptor antagonist SB-334867 (5 µM or 10 µM; n = 6 for each dose); group III (n = 6) received the non-selective (dual) ORX antagonist Almorexant (9 mM). On the day of the experiment, a dental needle (30-gauge; Mizzy, USA) was inserted until its tip was 0.4 mm below the guide cannula just before the experiment, and some minutes waited for habituation (the animal was already acclimatized, so it took only around 10 min). After habituation, the chamber was closed and remained closed until the end of the experiment. The microinjection was performed without manipulating the iguana to avoid stress. The needle was connected to a microinjection pump (model 310; Stoelting, USA) with a PE10 polyethylene tube (Clay Adams, USA) and a volume of 1 μL was injected for 30 s with a 5 μL Hamilton syringe. The dose and method of dissolving the drug were chosen based on pilot experiments and previous studies using SB-334867^15^, and Almorexant^67^.

The OX_1_R antagonist SB-334867 (Tocris, U.K.) was first dissolved in 4% DMSO (dimethyl sulfoxide) and then was diluted with 35% (2-hydroxypropyl)-β-cyclodextrin (Sigma-Aldrich, USA) in artificial cerebral spinal fluid (aCSF, pH 7.4 at 25 °C). For the vehicle, we used a solution containing 4% DMSO and 35% (2-hydroxypropyl)-β-cyclodextrin in aCSF. For the Almorexant (OX_1_R and OX_2_R antagonist, Cayman Chemical Company, USA), the same procedure was made, however, saline was used instead of aCSF. At the end of each experiment, 1 μL of 2% Evans blue solution was microinjected into the lateral ventricle. The animals were killed by pentobarbital overdose^68^. Upon dissection, we observed that the dye had diffused into the periventricular tissue and spread throughout the ventricular system, including the brainstem (Fig. 3G).

#### Experimental protocol for measurement of ventilation

One week after surgery, iguanas were placed in the experimental chamber at 25 °C for at least 6 h for acclimatization before the experiment. The chamber was continuously flushed with humidified air (1.5 L/min). Breathing was continuously recorded during the experiments. Baseline breathing (normocarbic normoxia, no injection) was recorded for at least 20 min and then the iguana received an i.c.v. injection of either the vehicles (groups I and II), SB-334867 (group III) or Almorexant (group IV). A 20 min interval was allowed to ensure full action of the drug and breathing to reach a “new steady state” before the iguana was exposed to hypoxia (5% O_2_, balance N_2_) or hypercarbia (5% CO_2_, 21% O_2_, balance N_2_) for 40 min. After the stimuli, animals were returned to room air (normocarbic normoxia) and breathing was measured during the recovery phase (1 h) before being exposed to the second stimulus. The order of the ventilatory stimuli was randomly. Preliminary experiments showed that this 1 h recovery interval is sufficient to allow breathing to return to baseline (pre-drug) while the drug is still active; the half-life of SB334867 is 4 h^69^ and of Almorexant is 32 h^70^. In each group, ventilatory measurements were performed both during the light (12:00–16:00) and the dark (08:00–12:00) phases of the displaced artificial light–dark cycle. Animals injected with vehicle were allowed to a second experiment in which it was used the other vehicle, randomly (Almorexant and SB-334867 had different vehicles). At least 1 week was allowed between experiments.

#### Data analysis and statistics

Ventilatory variables were analyzed by using software LabChart (version 7, ADInstruments). Calculations of respiratory variables were based on the last 10 min of the recordings [respiratory frequency (fR), tidal volume (V_T_), inspired ventilation (${\dot{\text{V}}}_{{\text{I}}}$), non-ventilatory period (T_NVP_), number of breaths per episode and frequency of episodes]. Segments of breathing traces were removed from the analyses when iguanas were active to ensure that breathing values used for the analyses were resting rates. During hypoxia and hypercapnia we analyzed the last 10 min period, which was chosen to include the most regular breathing pattern possible. This breathing pattern was chosen after ventilation reached the steady-state ventilatory response to hypoxia or hypercarbia. For the post-hypercarbic response, the calculations were based on the first minute after the beginning of the hyperpnea. The parameters in the graphs are displayed as percentage changes. The normocarbic normoxia values (baseline) represent the reference ones. Absolute values are shown in Table 1 (ventilatory parameters) and 2 (breathing pattern parameters). All values are reported as the mean ± s.e.m.

Breathing frequency was quantified by counting the number of respiratory events per min. Tidal volume was obtained from the integrated area of the inspired flow signal. Inspired ${\dot{\text{V}}}_{{\text{I}}}$ was calculated as previously described by^71^ as follows: ${\dot{\text{V}}}_{{\text{I}}}$ = V_T_ × fR. For the analysis of the respiratory pattern, the episodes were determined according to the criteria proposed by Kinkead and Milsom^72^ in which the number of breaths within an episode was obtained by counting the number of events which occurred in succession with no pause longer than the length of two events between them.

Statistical analyses were performed using GraphPad Prism (version 6.01, USA) and a P < 0.05 was considered significant. Variations of blood ORX-A along the day were determined using a one-way ANOVA followed by Tukey’s post- hoc test. To test the effect of the phase on the ventilatory variables it was used one-way ANOVA with Tukey’s multiple comparison test. Two-way ANOVA with Holm Sidak’s multiple comparison test was used to evaluate the effect of the antagonists on the respiratory parameters.

## Abbreviations

ALM: Almorexant
CNS: Central nervous system
CO_2_: Carbon dioxide
DAB: Diaminobenzidine
fR: Respiratory frequency
i.c.v.: Intracerebroventricular
LC: Locus coeruleus
MI: Microinjection
O_2_: Oxygen
ORX: Orexin
ORX-A: Orexin-A
ORX-B: Orexin-B
OX_1_R: Orexin receptor-1
OX_2_R: Orexin receptor-2
PBS: Phosphate buffer solution
PFA: Paraformaldehyde
RTN: Retrotrapezoid nucleus
rVLM: Rostroventrolateral medulla
T_NVP_: Non-ventilatory period
$${\dot{\text{V}}}_{{\text{I}}}$$: Inspired ventilation
V_T_: Tidal volume
